# High-fidelity endoscopic submucosal dissection simulator reproducing full-procedure training, bleeding, and perforation

**DOI:** 10.1055/a-2761-0309

**Published:** 2026-01-08

**Authors:** Takeshi Kanno, Yutaka Hatayama, Suguo Suzuki, Waku Hatta, Kaname Uno, Tomoyuki Koike, Atsushi Masamune

**Affiliations:** 1 38047 Division of Gastroenterology, Tohoku University Graduate School of Medicine, Sendai, Japan; 2 R & D Division of Career Education for Medical Professionals, Education Center, Jichi Medical University, Shimotsuke, Japan; 3 Division of Promotion for Gastroenterological Medical Innovation, Tohoku University, Sendai, Japan

Endoscopic submucosal dissection (ESD) requires advanced technical skills, including the ability to maintain stable visualisation and dissect within the submucosal layer while controlling bleeding and perforation 1 . Training is largely on-the-job, increasing safety and standardisation concerns 2 . Existing dry simulators allow basic incision and dissection practice but cannot reproduce critical complications or air-responsive luminal deformation 3 4 . To bridge these gaps, we developed a dry simulator capable of reproducing the full ESD sequence, including bleeding and perforation, within deformable stomach and colon lumens.

The simulator features a multilayered resectable lesion sheet mounted within a flexible lumen. The resin-based sheet reproduced the gastrointestinal tract layers including mucosa, submucosa, and muscularis propria. Sodium polyacrylate and polyethylene terephthalate fibres replicated submucosal lifting and tactile resistance. Simulated arteries were embedded in the submucosa to allow controlled bleeding 5 , while accidental deep incision exposed a yellow fat-like layer, effectively simulating perforation ( Fig. 1 , Fig. 2 ). The silicone-based airtight lumen, which deforms in response to air volume control, enabled realistic scope handling. The cardia and anal sphincter were modelled with thermoplastic elastomers to provide stable yet soft gripping, closely resembling real-patient procedures ( Fig. 3 ).

**Fig. 1:**
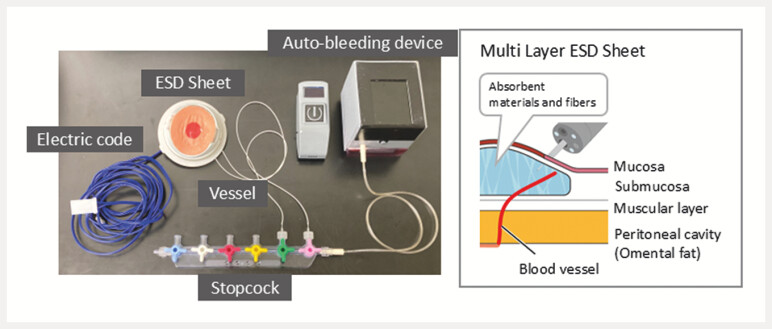
Structure of the multilayer resectable lesion sheet and associated complication reproduction. An overview of the endoscopic submucosal dissection (ESD) sheet that mimics mucosa, submucosa, muscularis propria, and an omentum-like adipose layer. The submucosa incorporates an absorbent polymer and fibres to reproduce submucosal lifting and tactile resistance during dissection. Artificial submucosal arteries permit controlled bleeding, and exposure of a yellow fat-like layer indicates perforation. Representative endoscopic views illustrate incision, submucosal dissection, bleeding, hemostasis, and perforation appearance.

**Fig. 2:**
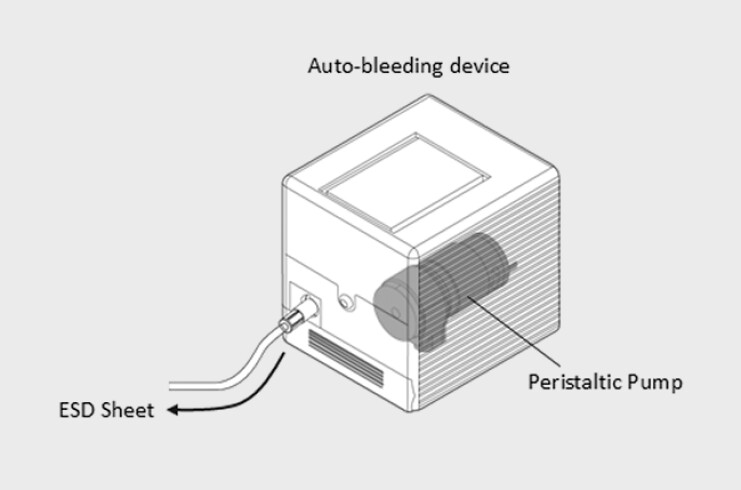
An automatic bleeding trigger mechanism. Demonstration of automated trigger for the reproducible onset of bleeding, used in standardised hemostasis practice.

**Fig. 3:**
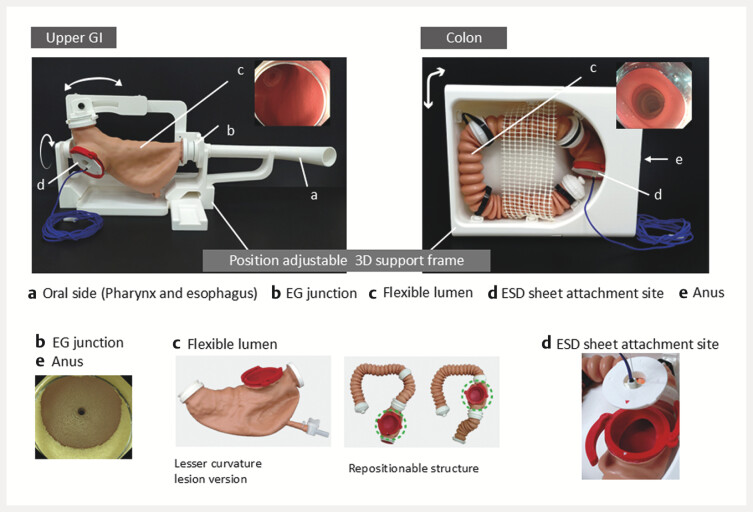
Upper-gastrointestinal and colon lumen models. The silicone-based, airtight lumens deform with insufflation and suction enabling realistic scope handling. The luminal pattern permits the adjustment of the lesion placement to reflect different anatomic configurations. The airtight design also enables observation under water and gel-immersion endoscopy. Representative images show deformation with air-volume control and lesion positioning in stomach and colon configurations.

Validation was performed on gastric antral lesions by five board-certified expert endoscopists between July and August 2025 ( Fig. 4 ). The median total procedure time was 25:42 (interquartile range [IQR] 22: 22–29:46) with low dispersion (coefficient of variation = 0.19). The median hemostasis time was 2:40 (IQR 2: 04–3: 52), with values clustering within 2 minutes of the median. Expectations for simulator-based learning rose following the trial compared to pre-trial levels. Free-text analysis identified the frequent co-occurrence of positive words such as “realistic,” and “useful” with procedural terms. A representative sequence of injection, dissection, bleeding, and perforation management is shown in Video 1 . This simulator provides realistic, reproducible simulation-based ESD training, promoting safer and more standardised education.

**Fig. 4:**
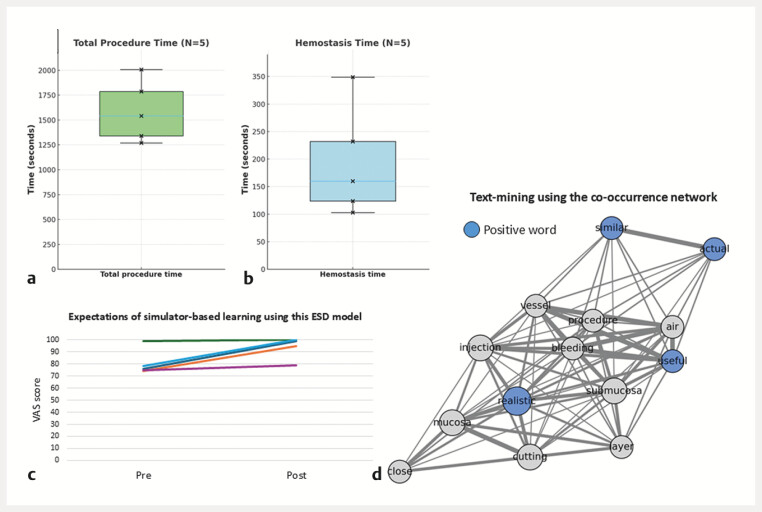
Expert validation of the novel ESD simulator. The pilot evaluation was conducted using the upper GI model. The resectable lesion sheet was affixed to the greater curvature of the gastric antrum, which is a common site for ESD practice. **a** Total procedure time ( *N* = 5). Boxplot with individual data points. Cross marks (×) indicate each expert’s score. The median total time was 25:42 (IQR 22: 22–29: 46; 1542 [1342–1786] sec), showing low variability (CV = 0.19). **b** Hemostasis time ( *N* = 5). Boxplot with individual data points. Cross marks (×) indicate each expert’s score. The median hemostasis time was 2: 40 (IQR 2:04–3: 52; 160 [124–232] sec). **c** Expectations of simulator-based training using this model. VAS scores improved after the procedure (median 76, IQR 74–78 at baseline; median 99, IQR 95–100 post-procedure). **d** Free-text analysis by the co-occurrence network. The node size indicates the document frequency, and the edge thickness reflects the Jaccard index. Only word pairs with a Jaccard index of >0.2 are shown. Blue nodes represent positive words identified in expert comments (e.g., realistic, actual, and useful). CV, coefficient of variation; ESD, endoscopic submucosal dissection; GI, gastrointestinal; IQR, interquartile range; VAS, visual analogue scale.

A simulated ESD procedure and its complications. An air-responsive flexible lumen; the multilayer sheet for the full ESD procedure (injection, incision, dissection, and excision); controlled bleeding visualised with gel immersion and Red dichromatic imaging (RDI); hemostasis, and perforation following muscular injury.Video 1

Endoscopy_UCTN_Code_TTT_1AU_2AB

## References

[1] Pimentel-Nunes P Pioche M Albéniz E Curriculum for endoscopic submucosal dissection training in Europe: European Society of Gastrointestinal Endoscopy (ESGE) Position Statement Endoscopy 2019 51 980 992 10.1055/a-0996-0912 31470448

[2] Zhang X Ly EK Nithyanand S Learning Curve for Endoscopic Submucosal Dissection with an Untutored, Prevalence-Based Approach in the United States Clin Gastroenterol Hepatol 2020 18 580 5880 31220645 10.1016/j.cgh.2019.06.008

[3] Coluccio C Jacques J Hritz I Simulators and training models for diagnostic and therapeutic gastrointestinal endoscopy: European Society of Gastrointestinal Endoscopy (ESGE) Technical and Technology Review Endoscopy 2025 57 796 813 10.1055/a-2569-7736 40185129

[4] Mitsui T Yoda Y Sunakawa H Development of new gastric endoscopic submucosal dissection training model: A reproducibility evaluation study Endosc Int Open 2022 10 E1261 E1267 10.1055/a-1845-5556 36118647 PMC9473824

[5] Kanno T Arata Y Hatayama Y Novel simulator of endoscopic hemostasis with actual endoscope and devices VideoGIE 2023 8 56 59 36820256 10.1016/j.vgie.2022.10.004 PMC9938294

